# Organising labour market integration support for refugees in Austria and Sweden during the Covid-19 pandemic

**DOI:** 10.1186/s40878-021-00264-y

**Published:** 2021-10-12

**Authors:** Almina Bešić, Andreas Diedrich, Petra Aigner

**Affiliations:** 1 Department of International Management, JKU Business School, Faculty of Social Sciences, Economics & Business, Johannes Kepler University Linz, Altenberger Straße 69, 4040 Linz, Austria; 2 Department of Business Administration, School of Business, Economics and Law, University of Gothenburg, Vasagatan 1, 41124 Gothenburg, Sweden; 3 Department of Sociology, Faculty of Social Sciences, Economics & Business, Johannes Kepler University Linz, Altenberger Straße 69, 4040 Linz, Austria

**Keywords:** Refugees, Covid-19 pandemic, Relational framework, Labour market integration, Labour market, Support organisations, Mainstreaming, Austria, Sweden

## Abstract

This paper addresses the question of how the Covid-19 pandemic has affected the labour market integration support (LMIS) organised for refugees in Austria and Sweden, and the potential consequences of the changes unfolding. LMIS for refugees is a complex phenomenon involving actors at different interwoven levels—the macro-national level, the meso-organisational level and the micro-individual level. However, the complexities and consequences of such processes for the labour market integration of refugees have so far received limited attention. The current Covid-19 pandemic actualises the need to gain a better understanding of how integration support is organised across the different levels and how the pandemic itself impacts such support. Thus, the article seeks to understand how the pandemic affects the LMIS organised for refugees in Austria and Sweden, two countries with a large refugee population and diverging responses to the pandemic. Based on 29 semi-structured interviews and three focus group workshops, the results highlight in particular three developments: (a) a further entrenching of broader, macro-national level developments related to integration support already underway prior to the pandemic; (b) further mainstreaming of activities; and (c) increased volatility of work. Overall, the pandemic has brought to the fore the interrelation of different levels in the organising of LMIS for refugees and has contributed to a stabilisation of already ongoing activities.

## Introduction

The pandemic will accelerate history rather than reshape it.

[*Foreign Affairs, 7 April 2020*].

This paper addresses the question of how the Covid-19 pandemic has affected the labour market integration support (LMIS) organised for refugees^1^ in Austria and Sweden, and the potential consequences of the changes unfolding. This is an important question to explore, for two reasons:

First, the labour market integration of refugees has over the past 2 decades become a crucial topic for policy makers and scholars (Bevelander & Pendakur, 2014; Fossati & Liechti, 2020) as employment is increasingly seen as pivotal for successful participation in society (Brell et al., 2020; De Vroome & van Tubergen, 2010; Feeney, 2000). At the same time, refugees face many barriers to entering the labour market. Attempts to explain these challenges have focused on their reasons for emigration, which are generally not to seek employment (Lee et al., 2020), their limited knowledge of the host country’s labour market (Delaporte & Piracha, 2018), their lack of access to relevant networks (Bevelander, 2011), their subjection to discrimination (Diedrich et al., 2011), and their health (e.g. Bogic et al., 2015).

A range of measures are in place in host countries to alleviate these difficulties and support refugees’ labour market integration, such as language tuition, guidance, preventive health work, work placements, mentoring programmes and a host of other activities (e.g. Battisti et al., 2019). Such support measures are usually organised by public, private and community organisations and volunteers, often in collaboration (see Ortlieb et al., 2021; Qvist, 2017).

The complexities and consequences of such organising processes for LMIS have only recently received more attention (e.g. Brorström & Diedrich, 2020; Diedrich, 2017). However, we lack knowledge of how the organising of LMIS for refugees has been affected by the Covid-19 pandemic, and the potential consequences for refugees and organisers of support.

Second, we compare Austria and Sweden as they each host a large number of refugees, with diverging welfare contexts (Esping-Andersen, 1990) and specific LMIS policies (see e.g. Konle-Seidl, 2018). The countries differ greatly in their responses to the Covid-19 pandemic. Whereas Austria mandated strict lockdowns as the number of Covid-19 cases increased, Sweden issued non-mandatory recommendations.

To study the changes unfolding in response to the pandemic, we lean on relational frameworks of organising (Syed, 2008; Syed & Özbilgin, 2009). We thus acknowledge LMIS for refugees as a dynamic, multilevel process connecting the macro-national, meso-organisational and the micro-individual levels in mutually constituting ways (Knappert et al., 2020; Lee et al., 2020).

Previous research has pointed to numerous difficulties for refugees at each of these levels (e.g. Fasani et al., 2018). Early investigations suggest that some of these challenges have been further exacerbated by the Covid-19 pandemic (Guadagno, 2020). However, we argue that the emerging narrative of exacerbated difficulties for refugees as a result of the pandemic, which is based on their comparison to other crises (Takenoshita, 2017), must be expanded to take into account the interrelated and interdependent multilevel challenges refugees face in the host countries’ labour markets in light of changes in the provision and organising processes of integration support. In doing so, the article points to three important consequences from the changing relationships in the context of LMIS across the two countries: (a) broader, macro-national level developments already underway prior to the pandemic become further entrenched; (b) mainstreaming practices increase; (c) the increased volatility of work affects refugees directly and indirectly.

In the following, we present an overview of the scholarly work on LMIS for refugees and a relational framework as a meaningful tool to analyse the unfolding changes to LMIS during the pandemic. We then illustrate the backdrop against which LMIS for refugees is organised in Austria and Sweden and recent developments in response to the pandemic, before outlining our methodology and presenting our findings. Finally, we conclude with a discussion of the implications of our findings.

### Emerging challenges of LMIS for refugees

Refugees face many challenges in their efforts to enter the labour market (e.g. Fasani et al., 2018; Lee et al., 2020) and are often deemed to require special support. A range of studies have explored these challenges from a policy perspective, by focussing on asylum or labour market policies and their impact on labour market integration (e.g. Emilsson, 2015; Hagelund, 2020; Jutvik & Robinson, 2020). Other research addresses obstacles affecting refugees at the individual level, e.g. language difficulties, health problems, or a lack of networks (Barslund et al., 2017; Verwiebe et al., 2019). Research on how support for refugees is organised in practice, involving a plethora of organisations, is limited, with few exceptions (see e.g. Ortlieb et al., 2021; Brorström & Diedrich, 2020).

However, the challenges connected to LMIS are interrelated, and in practice it is difficult to separate them. On the one hand, a change in policy may have consequences for both organisers of support and refugees. On the other hand, challenges associated with refugees as a particular group of new arrivals, such as the fact that they suffer more from health problems, may result in changes in policy. Further, changes in organising practices, such as the introduction of new communication technology, may increase obstacles such as language difficulties on the individual level. Yet few studies have highlighted how the practical work on integration support for refugees is multifaceted, with challenges emerging through the shifting relationships between these different levels. One way of capturing these relationships and challenges is through a relational perspective (Syed, 2008; Syed & Özbilgin, 2009). Exploring the field of diversity management, Syed and Özbilgin (2009) argued that many studies over-emphasise the policy level at national or organisational level, and through such “single-level conceptualisations” neglect the interplay of multilevel challenges and structural and intersectional concerns of diversity and equality. Instead, they suggest, the macro, meso and micro domains of diversity exist in a state of relational interdependence, as individual workplace experiences and perspectives are not just a product of, but also contribute to, the macro and meso responses towards diversity (Syed & Özbilgin, 2009).

Drawing on a relational perspective, it is essential to understand how broader societal issues at the macro level affect the organising of LMIS. Meso-organisational factors have a direct role in determining and influencing the integration of refugees into the labour market. On the micro level, the activities and events involving refugees and others influence integration into the labour market. We argue here that the roles policy makers, organisations and individuals can play in effectively organising LMIS are influenced by issues and changes at multiple levels.

Such a perspective also helps in understanding how refugees not only interact with, but also enact, environments and utilise their own resources to shape their future in the host economy (e.g. Hunt, 2008). Their attempts at integrating into the labour market should at the same time be seen as influenced by macro-national and meso-organisational opportunities and barriers. In other words, macro-national and meso-organisational phenomena are a result of—and are in turn influenced by—micro-individual activities, with potentially important implications for individual-level opportunities and outcomes.

By using a relational framework, we are able to address micro-individual, meso-organisational and broader macro-national issues together by pointing to the importance of their mutually influencing relationships based on contextual, contingent and comparative elements that allow them to be situated in their social and historical contexts (Syed & Özbilgin, 2009).

### Austria and Sweden: organising LMIS in light of Covid-19

Both Austria and Sweden have large migrant populations and have, in recent years, admitted more refugees per capita than most European countries. In Austria over 88,000 individuals applied for asylum in 2015 while Sweden accepted close to 163,000 asylum seekers (Eurostat, 2020). These numbers have dropped markedly since then, in response to the 2016 EU-Turkey deal and the introduction of restrictive asylum and migration policies in both countries. In 2019, around 12,000 asylum seekers arrived in Austria of which 6,000 were granted refugee status, and 26,000 asylum seekers arrived in Sweden, where 5,700 received refugee status (Eurostat, 2020). In both countries, refugees continue to face many challenges to become an integrated part of society (e.g. Verwiebe et al., 2019; Wikström & Sténs, 2019). Below, we outline the organisation of LMIS showing the move towards mainstreaming at the policy level as well as differences in e-Governance and responses to the pandemic, which impact the provision of LMIS for refugees.

### LMIS in Austria and Sweden

In Austria, the Integration Act and the Labour Market Integration Act, both passed in 2017, form the basis for labour market integration. The laws distinguish between asylum seekers with a high probability of being allowed to stay, and refugees and beneficiaries of subsidiary protection. The latter two groups are obliged to sign an integration contract and participate in an ‘Integration Year’. The Integration Year bundles existing labour market measures, focusing on competence, language courses, values and orientation, and job preparation measures (Ortlieb et al., 2021). The main organisers of LMIS are the Austrian Integration Fund (AIF), which manages the Integration Year, and the Austrian Public Employment Service (APES). AIF and APES cooperate with several non-governmental organisations (NGOs) and other private service providers in delivering LMIS measures across the country. Additionally, municipalities implement projects and support measures that aim to complement the AIF/APES measures. After the Integration Year, refugees have full access to the job market and to the general services provided by the APES.

In Sweden the legal basis for labour market integration is the Act on the Responsibility for Settlement Measures for Recent Immigrants. The Act stipulates that refugees granted a residence permit for protection and received by a municipality shall have the opportunity to participate in the 2-year Settlement Programme run by the Swedish Public Employment Service (SPES). As part of the programme, refugees are offered a range of measures to facilitate their integration into the labour market, such as subsidised employment, skills assessment, language training, civic orientation courses, education, vocational training, internships and matching activities. The SPES does not undertake these activities itself. Instead, LMIS for refugees is decentralised, and involves a plethora of public, private and community organisations, including companies, municipalities and other state agencies and non-profit organisations (Diedrich, 2017; Diedrich & Hellgren, 2018; Qvist, 2017).

Additionally, LMIS in Sweden has been influenced by broader movements within the Swedish public sector towards e-Governance (e.g. Bernhard, 2014; Garcia Alonso et al., 2020), not least visible in the 2015 establishment of the eSam programme around digitalisation that involves 28 national and local authorities. The country is ascribed a fast-growing level of “digital maturity” and its citizens today communicate predominantly with authorities via digital channels (Swedish Center for Digital Innovation, 2020). Influenced by these developments, the SPES began to replace face-to-face contacts with jobseekers with digital communication tools—even before the onset of the pandemic. In contrast, in Austria the digitalisation of the public sector progressed very slowly. While some efforts towards e-Governance had been made, APES and other organisers of LMIS have only recently started to broadly implement digital tools in practice (Bröckl & Bliem, 2020). APES, for instance has begun to translate its digitalisation strategy into practice, by financing courses to increase the digital skills of trainers in support organisations.

Notably, the restrictive asylum and migration policies introduced in both countries after 2015 have led to a successive decrease in the numbers of refugees in the Integration Year and on the Settlement Programme, as well as in other support programmes. At the same time, agencies and public, private and non-profit service providers in both countries developed an array of measures specifically focussing on asylum seekers and refugees. In line with broader moves towards mainstreaming of labour market integration policies from 2018 onwards, a mainstreaming of such measures can be observed in practice, where refugees are to be treated first and foremost as jobseekers. Subsequently, much of the specific support available to refugees before 2018 is no longer available today.

### Responding to the Covid-19 pandemic

In response to the pandemic, the Austrian government imposed the first lockdown in mid-March 2020, which led to widespread closures of businesses. After a brief re-opening during summer 2020, two subsequent lockdowns were imposed, in November 2020 and December 2020, and only a few restrictions were lifted in February 2021 (Böheim & Leoni, 2020). The pandemic continues to impact the labour market, with unemployment figures standing at around 10% in February 2021 (APES, 2021). The Austrian job market is furthermore faced with a significant number of furloughed workers (around 300,000) and a 7% drop in GDP for the first few months of 2021 (WIFO, 2021).

Sweden’s response to the Covid-19 pandemic differed from most other countries, including Austria. Until January 2021, the Swedish government introduced mainly non-mandatory hygiene and physical distancing recommendations in the hope that people and organisations would follow those (Kavaliunas et al., 2020). The Swedish strategy of working mainly with recommendations was widely seen as an expression of the public’s strong trust in the country’s government and public institutions. Most stores, hotels and restaurants remained open throughout the pandemic and the Swedish economy has not suffered to the same degree as other European countries. Nevertheless, the labour market was impacted, with unemployment standing at 8.3% in September 2020 and a record number of furloughed workers: 71,740 by November 2020 (Swedish Agency for Economic & Regional Growth, 2020). Unemployment is expected to increase to over 11% during 2021 (Swedish Public Employment Service, 2020).

In both countries, public institutions closed and state agencies such as APES and SPES mandated their employees to work from home and avoid face-to-face meetings with clients. Communication in general was instead facilitated digitally or via telephone (during lockdowns in Austria, but throughout the period in Sweden).

This brief overview of LMIS, and the developments in response to the pandemic, shows that the policy direction in both countries appears to move towards more mainstreaming (Scholten, 2020; Scholten et al., 2017) in labour market integration. We argue that the pandemic has further accelerated this development. Additionally, while the responses to the pandemic are diverging and the digital maturity of the public sector differs, we argue that the Covid-19 pandemic has impacted the actors involved in LMIS in similar ways, as outlined in the findings section further below.

## Methodology

We applied a qualitative methodology, based on (1) an examination of relevant documentation in the analysed countries, and on (2) fieldwork through semi-structured interviews and focus group workshops. This approach is well suited for the exploration of under-researched topics, such as the impact of Covid-19 on refugee labour market integration. At the same time, we respond to calls for more comparative research in the field of refugee integration (Bevelander, 2020; Bevelander & Pendakur, 2014).

In both countries, we relied on semi-structured interviews with refugees where possible, and with organisers engaged in LMIS.

Two sets of interview questions were developed for the organisers and the refugees respectively. Those for the organisers were constructed with the emphasis on the investigation of measures for refugees before and after the Covid-19 pandemic, focusing on the view of organisers and institutional perspectives. The interview guide for refugees was constructed with a focus on the individual perception of support measures and the overall integration processes in the labour market. How the support for refugees changed due to the pandemic was specifically investigated.

In order to reach both organisers and refugees, different sampling strategies were employed, to take into account that access to refugees was difficult. Consequently, deductive theoretical construct sampling and snowball sampling were utilised (Patton, 2015). Gatekeepers, who control access to the information, were contacted and helped to reach out to refugees and experts. These included NGOs, state agencies and other institutions involved in LMIS. During the research process, as numerous difficulties were encountered in accessing respondents, snowball sampling proved increasingly meaningful in improving access and enlarging the number of interviews.

Table 1 gives an overview of the interviews conducted in Austria (15 interviews) and in Sweden (14 interviews). Additionally, in Sweden during November and December 2020, three focus group workshops (Wilkinson, 2004) were undertaken with representatives from companies, public sector organisations and volunteer/non-profit organisations allowing us to gain further insights into the LMIS for refugees.

**Table 1:** An overview of interviews conducted

Country	Interview partner	Stakeholder	Interview
AT	R1 (Recently employed)	Refugee	Online and via phone
	R2 (Job seeker)		
	R3 (Job seeker)		
	R4 (Student)		
	R5 (Job seeker)		
	R6 (Job seeker)		
	R7 (Job seeker)		
	O1 (Handling officer)	Private support org. 1	
	O2 (Project manager)	NGO1	
	O3 (Handling officer)	Private support org. 2	
	O4 (Handling officer)	NGO2	
	O5 (Handling officer)	NGO3	
	O6 (Trainer)	NGO1	
	O7 (Social worker)	NGO1	
	O8 (Integration officer)	APES	
SE	Handling officer 1	SPES	Online, via telephone and face-to-face
	Partner organisation		
	Manager 1 (twice)		
	Handling officer 2		
	Handling officer 3		
	Handling officer 4		
	HR specialist	Large bank	
	Project leader 1	Initiative for entrepreneurs	
	Project leader 2	Municipality 1	
	R1 (Job seeker)	Refugee	
	R2 (Job seeker)	Refugee	
	R3 (Job seeker)	Refugee	
	R4 (Job seeker)	Refugee	

*R*, Refugee; *O*, Organiser

The interviews in Austria were conducted solely online, or via telephone. In Sweden, four interviews were conducted face-to-face, while the remaining interviews were conducted online and via telephone. All were conducted between May 2020 and February 2021, and lasted between 30 and 110 min, were digitally recorded, transcribed and analysed with MAXQDA (Austrian data) or manually coded (Swedish data).

The qualitative data analysis followed a thematic analysis (Braun & Clarke, 2006) through coding of the interview transcripts. The process was ‘data driven’ with the aim of identifying themes in the support of labour market integration for refugees and the impact of the pandemic. In the following section we present our findings along the lines of the three broad themes identified in relation to the changes in LMIS for refugees in response to the pandemic.

## Findings

### Entrenching and fostering macro-national developments toward digitalisation

The first important developments in LMIS due to the pandemic are connected to changes in how organisers of integration support and refugees interact in Austria and Sweden. These changes are a result of the regulations put into practice in both countries to curb the spread of the virus—Austria’s series of tough lockdowns and Sweden’s “recommendations”. Not surprisingly, personal contacts ceased in Austria, but in Sweden, too, personal contacts were greatly reduced, in particular in the public sector, which was urged to set an example for the rest of society. In the context of LMIS for refugees, contacts were increasingly facilitated through the use of digital and other communication tools. In Austria, these changes happened as the public sector was slowly moving towards greater use of digital technology in its operations. Our material indicates that the organisations were hesitant to adopt digital technology in their daily work, perceiving digitalisation as more of a burden than an opportunity. However, when the pandemic hit, severe restrictions forced APES and other support organisations to facilitate contact with the refugees via digital technology:

Only then [after the pandemic hit] have we created this infrastructure, and we have started to deal with this question of how to do things online. (AT_O4_NGO2).

In Sweden, different developments were observed. In line with the ongoing focus on digitalisation, the SPES had been working since 2018 to replace personal contact with jobseekers, including refugees, with digital communication wherever possible. Information campaigns describing the advantages digital communication offered to jobseekers were run through multiple channels. Refugees in particular, whom handling officers described as “less digitally literate compared to native Swedes”, were informed about the possibilities of handling the compulsory reporting of activities on the Settlement Programme, for example, via their computers or mobile phones. They were also given mandatory tasks to practise such reporting. These efforts to “digitise” refugees were accelerated by the Covid-19 pandemic. As one senior SPES manager working with refugees explained:

All spontaneous visits are kept to a minimum. People may cross the threshold, but have to leave again immediately [laughs]. The only positive thing is, if one can see it as positive at all, that [the pandemic] has necessitated an increased digitisation among our jobseekers. That’s something we’ve battled with for many years—that whatever you can do yourself, you should do yourself. But now they’ve been forced [to do it], because there's no other way to get help. That’s, well, something positive that has come out of all this. (SE_SPES handling officer).

The digitisation of refugees is here seen as positive and a necessary element in the efforts to make the SPES more efficient through digitalisation and a move towards e-Governance. Public officials hence made sense of the pandemic as an opportunity to push changes in practice that had already been underway before the pandemic.

In Austria, the data results of the study indicated the increasing importance of digitalisation *because of* the pandemic. Some support organisations considered adapting their measures to include more digital skills support, although this is in its infancy as explained below:

I think during corona this [digital inclusion] has received more attention. […] The topic of digital competences and how to deal with specific marginalised groups will also become more important in the sense of getting funding from APES or ministries. But currently there’s a big need there, and due to corona the discussion about this is happening much, much faster. I hope it doesn’t get lost among the other big topics on the labour market, because this is about low-threshold measures regarding digital competence support for these groups. (AT_O5_NGO3).

As several support organisations return to personal support in a smaller capacity, the potential boost for digitalisation is proving to be a rather slow process in Austria.

How digitalisation has affected people on an individual level varies, of course, greatly. However, we have identified some important commonalities in both countries. In Sweden, the handling officers at the SPES pointed to an increasing frustration among refugees with the authority’s decision to facilitate all communication via digital channels. Our material suggests that the degree of frustration may be correlated with how well an individual understands the Swedish system and context and is able to make decisions related to his/her integration independently. According to the handling officers, refugees who expressed the need for more help tried more often to meet them in person, whereas those described as more independent and confident demanded fewer personal contacts. Digitalisation also seems to have opened up a digital divide (Blix, 2017) between educated and less well-educated refugees and between younger and older (notably 50 +), with the latter experiencing greater difficulties in dealing with online job applications or online training provided by support organisations:

There are a lot of people who are […] unable to handle email or send attachments […]. And now that the applications are sent online […] they are excluded from the process. And that’s especially difficult for the people who are less qualified. It used to be like this: I simply took my resume […] and said, I need a job and they said “Okay, I'll get back to you when we have something.” Now that works too, but you have to upload the resume online. That’s when some people fail. […] It will certainly continue to be an issue, because we have seen how much the job landscape has actually changed. In this low-skilled area too. Maybe it was already like that before, but we didn't have it on our radar. (AT_O4_NGO2).

Our findings from the interviews and focus group workshops suggest that the move to digital communication has negative effects on the refugees. As highlighted in the focus group workshops, notably, they usually lack personal and professional networks, deemed important for integration, but more difficult to cultivate when people cannot meet physically.

### Further mainstreaming of LMIS

The second major development concerns changes in policies in both countries that had been underway before the pandemic. One APES representative explained:

[…] in 2018, as you have surely seen in the media, we had budget cuts. Especially in integration measures for refugees. [Before] we had a variety of options. We had a special fund with a [separate] budget. There we could support them [the refugees] individually. And a lot has changed there, where we had to limit some of our support offers. (AT_O8_APES).

The pandemic seems to have amplified such mainstreaming practices as funds were directed towards “the whole of society” (Scholten et al., 2017) as specific measures targeting refugees were phased out, temporarily paused or merged with activities for supporting other groups of job seekers. As one NGO representative from Austria explained:

[…] From 2020 onwards, […] the project has simply been extended to include people with a migration background in general (AT_O2_NGO1).

Government responses to the pandemic in both countries did not prioritise refugees, and no additional resources were allocated to support them into employment. On the contrary, our material suggests that in some cases resources intended to support refugees were redirected towards helping jobseekers who had recently lost their job due to the pandemic, as highlighted by the APES representative:

Yes, my impression is that due to the pandemic refugees are not a core issue any longer. Either this target group is less in the spotlight politically, because [the pandemic] affects everyone, or the integration is running well […] or the political will is not there. So several factors could play a role. But we see a change; as I said, since 2018 there have been restrictions [in funding] in the integration measures […] (AT_O8_APES).

Subsequently, there was a strain on support organisations implementing measures aimed at refugees in particular, as it became unclear if projects would be extended at all, and if they were extended, what their focus would be:

But, […] what next year will be like, I don't think the [APES] is aware of that yet. It always depends on the funds they have available. (AT_O1_private support organisation1).

Those refugees that finish the compulsory Integration Year in Austria have little additional support. At APES they can access general support measures offered to all job seekers. The pressure on refugees to act as “regular job seekers” increased as well:

Currently the APES puts a lot of pressure on these people. They are expected to find work faster, even if they don’t speak German they are supposed to send out applications. But, they cannot do that. We support [them] as much as we can in writing job applications, but really the pressure from the APES is currently extreme. […] We’ve never had a time like this. This has grown. Since the Corona crisis, these people get numerous jobs that they have to apply for […]. If they don’t fulfil this, then there are sanctions. At times the APES checks this with the companies, i.e. if they have really applied for a job. […] So, put like that, there is a lot of control from APES (AT_O7_NGO1).

In Sweden too, mainstreaming of LMIS could be witnessed in connection with a range of reforms the SPES had gone through since 2014 aimed at increasing the efficiency of the agency. Subsequent reductions in the number of handling officers during 2019 meant that the remaining handling officers had less time to support each jobseeker and could no longer specialise in working to help refugees within specific occupations or based on their particular skills:

Every person who has had a somewhat deviant situation has been negatively affected. […] when there’s something that is a bit outside of what we [handling officers] do all the time now […] We previously had the advantage that if you did something all the time you were able to keep yourself updated about the changes that occurred […] For example, someone with a background as a teacher […] for a handling officer working only with highly-skilled people, it wasn’t difficult to know how to proceed to support that person. (SE_SPES Handling officer 4).

Additionally, the continuous mainstreaming of measures led to a reduced number of handling officers who were now faced with an expanding group of jobseekers. Less time allocated for each individual meant that any person falling outside of “the norm” was affected negatively, as one Swedish public official explained:

Support is much more limited. For example, previously, people who were illiterate or who weren’t very good at using computers could get at least some rudimentary help with their digital needs […] for example help with reporting their activities. Even if we didn’t give them back [the old] paper documents to fill in, we could still help by showing them how to do that digitally, so that they could learn how to do that. We can’t do that anymore now because of corona. (SE_SPES Handling officer 4).

The challenges arising for refugees and organisers through the redirection of funding and other resources away from LMIS for refugees were compounded by the pandemic. Communicating digitally and over the telephone, for example, could lead to an increased workload for the organisers as they felt they were losing control of the job seekers:

There was a need there to follow up with the participants after a training course. Colleagues—they often called afterwards and asked how he was doing, how she was doing, and do you know anything about him, and why wasn't he at the zoom meeting? (AT_O1_private support organisation1).

The pandemic and resulting shift to digital communication also affected some support activities that had been specifically designed with certain target groups in mind. A SPES official, in charge of an integration support initiative financed by the European Social Fund in Sweden lamented:

I work for a project run by the SPES which aims to facilitate long-term matching [between refugee women and employment opportunities]. We use a method, which we are currently testing, “Matching from Day 1”. That method has previously proven to work well for women with short educational backgrounds, to ensure that they can become employed to the same extent as men. When the pandemic hit, we couldn’t continue working with the method. It’s very much about meeting physically in what we call empathetic spaces, about building relationships and about getting to know the person. It’s equally about getting to know the employer and talking about what kind of person they are looking for. […]. The third step then is about accompanying the jobseeker to an interview with the employer. Because we know both parties, we help them in their meeting so that we don’t end up with an awkward blind-date situation. We can’t do any of that now. (SE_SPES caseworker and project leader).

The examples above show activities that intentionally began to target broader groups than refugees before the pandemic (mainstreaming), but where these were compounded by further shifts—many unintentional—away from refugees towards the “whole of society” because of the pandemic. This shift has been fostered through social distancing as well as budget cuts or redistribution of funds. Notably, initiatives that offered specialised support beyond the “normal” support for refugees, because they were forced to adapt their activities to adhere to the recommendations of social distancing, experienced what could be described as “de-specialisation” as particular forms of personal interaction were replaced by communication via digital tools.

### Increased volatility of work

While Austria and Sweden implemented different response measures, the pandemic impacted both economies and labour markets in major ways. Higher unemployment levels meant fewer opportunities for refugees to enter the labour market, as one handling officer in Austria explains:

We have requirements from the APES that we must place a certain percentage of our employees on the labour market […]. And that’s of course the big challenge, especially in these times of high unemployment […]. And I would say that what used to be an approach for us, the retail and catering industries, are rather reluctant to enter the market. And that’s something we feel. […] At the moment the job market isn’t receptive. (AT_O3_private support organisation2).

And refugees have to compete with an increasing number of other jobseekers:

Many more clients [apart from refugees] that we advise through our measures are actively looking for work, because they lost their [job due to the pandemic]. (AT_O5_NGO3).

The pandemic-related lockdowns even put jobs that had been thought of as secure at risk, as one refugee explained:

It is currently very difficult, because many companies do not even take new employees due to corona. You also don't get a trainee position, or it is postponed, after the first lockdown, then the second, and now the third. For example, I am still waiting for an apprenticeship in [an organisation], but I did not get the confirmation because the third lockdown was prolonged. Now I have to wait even longer until it finally starts at some point. (AT_R6).

In Sweden too, higher unemployment levels meant it was more difficult for refugees to enter the labour market. Summer jobs, for instance, widely seen as a meaningful first step for refugees to enter the labour market, had all but disappeared, as one refugee explained:

As a student, I found it very difficult when I wanted to apply for a summer job this year (2020). Few positions were available. I worked as an assistant nurse in an elderly care home for a longer period of time, but this year so many elderly care homes reduced their staff and did not offer any summer jobs (SE_R1).

The short-fall in entry-level jobs was seen as a major obstacle for refugees’ labour market integration, as one representative of a volunteer organisation in Sweden explained:

We see that our participants [refugees] on the various programmes are affected really badly. Young people outside of the labour market are not getting their first important jobs, for instance in the service sector, and run the risk of falling into the category of long-term unemployed in the future. And all those people who come to us, who worked as in-house consultants at Volvo have been hugely affected due to all the redundancies. And we see that our participants [refugees] face an extremely tough situation. (SE_Notes from focus group workshop).

Jobs attractive to refugees fell away, and they were increasingly employed on short-term contracts. Here, too, changes due to the pandemic contribute to country-specific developments already underway: in Austria, the increasingly important role of temporary staffing agencies in employing refugees, and in Sweden, the use of subsidised forms of employment financed by the government when employing refugees. In Austria, our data suggests that companies have become more cautious in their hiring decisions. Additionally, many companies that traditionally employ large numbers of migrants (including refugees), such as the service and hospitality sectors, have furloughed many of their workers and are not hiring new ones, as explained below:

[The] Corona situation has a very strong impact on the economy, on many companies, simply where production and export slumps are. And that’s not including the hospitality sector, where a lot of jobs have been lost, and these are often entry-level jobs for our clients. (AT_O5_NGO3).

The cautious approach by many companies in taking on new staff has given a boost to temporary staffing agencies:

This is one thing I notice first and foremost, that many job advertisements are through staffing agencies. […] there are fewer companies that are now willing to take on new staff. And it has also become a bit more difficult for people with a migration background to find work, because many people who have been in Austria for a longer time, whether they have a migration background or are locals who have lost their jobs due to corona, are now looking for a job. Yes, and these people then have better chances than those who have only been in Austria for 4–5 years, and perhaps they are only gaining their first professional experience here in Austria. These people are naturally finding it more difficult now. They have a disadvantage here. (AT_O2_NGO1).

In Sweden, similar evidence suggests that some employers are pausing their initiatives and projects supporting refugees into employment. Others, mainly smaller companies affected by the pandemic, are employing refugees on a larger scale on short-term, state-subsidised contracts. Given the negative effects of the pandemic on sectors employing many refugees, the increase in subsidised, short-term employment contracts for refugees within these sectors can be seen as further entrenching more precarious employment situations in the longer term.

While some of these findings are in line with previous studies (e.g. Falkenhain et al., 2021; Vogt, 2019) our material points to an additional challenge embedded in the relationships unfolding as part of LMIS: The increasing volatility of work appears to affect not only refugees, but also the organisers of LMIS. In Austria, case workers are faced with an uncertain future in the support organisations:

This is also a huge uncertainty for me and for my colleagues as well. Suddenly it can really mean […], the project was not funded or it was shortened again. And yes, you have to start searching [for work], so to speak. (AT_O2_NGO1).

And a representative of a Swedish volunteer organisation explained at a workshop:

During crises, people want to help others. We saw that in 2015 [the year of the refugee crisis]. However, now things are different. The entire labour market has been turned upside down, and potential volunteers who previously had secure employment, don’t have that anymore. (SE_Notes from focus group workshop).

This suggests that the volatility of work not only affects refugees directly, because there are fewer jobs available to them, but may also indirectly affect their integration into the labour market as it influences support activities, because volunteers are more difficult to find and other staff at support organisations might have to be let go. This can have longer-term consequences for the labour market integration of refugees, not least in terms of mainstreaming.

## Discussion

There is little doubt that the ongoing Covid-19 pandemic is experienced as a dramatic event in both Austria and Sweden, igniting further processes of change in most spheres of public and private life. Our question was how the pandemic influenced the provision and organising processes of LMIS for refugees in these two countries. Leaning on a relational framework (e.g. Syed, 2008; Syed & Özbilgin, 2009) has allowed us to describe some of the important relationships between the macro, meso-organisational and micro/individual levels, thereby providing a counterpoint to the vast number of studies of labour market integration of refugees that approach the phenomenon by focussing either on a macro perspective, predominantly emphasising labour market economic variables (e.g. Bevelander & Pendakur, 2014), an organisational perspective with a focus on particular organisations involved in labour market integration of refugees (e.g. Ponzoni et al., 2017), or an individual perspective often focussing on the particular predicaments of individuals or groups (e.g. Bucken-Knapp et al., 2019). The relational framework instead directed our focus onto the interplay of multilevel challenges (e.g. Knappert et al., 2020) as well as structural and intersectional concerns of the provision of LMIS for refugees, allowing us to yield a number of important theoretical and practical contributions:

First, notwithstanding varying responses to the pandemic in different countries, the labour markets in both countries seem to have been affected similarly, with record unemployment levels and numbers of furloughed workers. As has been shown in previous studies, such economic downturns tend to affect vulnerable groups on a larger scale than other groups (Vogt, 2019). Our material points to the apparent difficulties for refugees in finding employment. However, while previous studies (Papademetriou & Terrazas, 2009) have mainly offered quantitative, single-level conceptualisations of changes in employment levels for refugees during crises and economic downturns, we have—by implementing qualitative methods—captured some of the interplay of multilevel challenges as well as structural and intersectional concerns of refugees’ labour market integration, additionally giving individual players acting in those settings a voice. We show that the macro-national, meso-organisational and micro-individual domains of LMIS exist in a state of relational interdependence, because activities, experiences and perspectives on each level are not simply a product of, but also contribute to, the activities, experiences and perspectives on the other levels. Thus, mainstreaming practices promoted by restrictive asylum, migration and labour market policies instituted prior to the outbreak of the pandemic may be further entrenched and stabilised when digital technology is employed as a response to social distancing regulations in a way that leads to what we refer to as a *de-specialising* of activities organised for refugees. While a trend of mainstreaming policy in the area of integration has been visible for some time across Europe (Scholten et al., 2017), which can be seen as a development towards acknowledging ‘superdiversity’ in some European cities and regions, we show some challenges emerging from such an approach when it comes to vulnerable groups. Specifically, as our findings show, in the area of LMIS, mainstreaming—particularly in times of crisis—can have repercussions for already vulnerable groups such as refugees, and for the organisations supporting these groups. Thus, in some cases targeted policies might contribute to the reduction of inequality, whereas mainstreaming can reinforce inequality (see also Scholten & van Bruegel, 2018; Phillimore, 2012). Furthermore, mainstreaming over time cannot be seen as a single-level concept, whereby a policy is implemented top-down. Rather, it is more meaningfully understood as translated into practice in a multidirectional, multilevel and mutual process unfolding over time, with intended and unintended consequences.

Second, while crises have been seen in the past as events where institutionalised practices and structures are destabilised, our study shows a somewhat different picture: In Austria, established practices were indeed destabilised as digital tools needed to swiftly replace face-to-face contact in the absence of an advanced eGovernance policy. Subsequently, only a necessary minimum in terms of digital communication tools was implemented with the aim to revert back to in-person communication. In Sweden, on the other hand, developments already underway before the pandemic were stabilised as part of the responses to the pandemic. For example, the increasing use of digital communication tools in the Swedish public sector during the pandemic has contributed to further entrenching the government’s e-Governance strategy, already commenced with vigour before the pandemic.

Third, our findings give some indication that the increase in volatility of work as a result of the pandemic affects not only the refugees, as some early studies have indicated (e.g. Falkenhain et al., 2021), but also the organisers of integration support activities. Our relational perspective permits us thus to point to possible difficulties refugees face in entering the labour market in the longer run as an indirect result of support staff being furloughed and the subsequent absence of, for example, volunteers. This may indirectly amplify the negative effect on refugees, as the discontinuation or downsizing of initiatives due to lack of funding or lack of volunteers can mean a further complexifying of refugees’ opportunities to build or access business and personal networks, seen as vital in facilitating their labour market integration (Bevelander, 2011). Additionally, the pandemic seems to have intensified the trend for hiring workers through temporary staffing agencies and on short-term contracts. As competition in the labour market increases, this is bound to negatively impact refugees who are already at a disadvantage when looking for work. The government policies in Austria and Sweden seem to focus on individuals who have recently lost their jobs due to the pandemic and are deemed to stand a good chance of finding new employment. The long-term unemployed, and this includes refugees, are not prioritised, meaning that no additional resources are currently allocated to helping this group into employment (e.g. Frödin & Kjellberg, 2020).

To conclude, the pandemic has led to profound changes in economic and social life in both Austria and Sweden. We have shown here that some of these changes in the context of the organising of LMIS for refugees were not initiated as a response to the pandemic but were already ongoing, only to be amplified through the pandemic response. These developments do not seem to have disrupted LMIS for refugees in either country, but they do seem to have pushed it further in specific directions. Looking forward, we point to the possibility of a gradual stabilisation of the changes over time, in particular when they become enacted not as responses to the pandemic, but as a continuation of already established approaches to LMIS for refugees.

## Data Availability

Further information on the data used and/or analysed during the current study are available from the corresponding author on reasonable request.

## Footnotes

1 We view* refugees* in the broadest sense as individuals who have fled their home country and who have either applied for or have already been granted international or national protection.

## References

[CR1] APES. (2021). *Arbeitsmarktdaten*. Retrieved March 11, 2021, from https://www.ams.at/arbeitsmarktdaten-und-medien/arbeitsmarkt-daten-und-arbeitsmarkt-forschung/arbeitsmarktdaten.

[CR2] Barslund M, Busse M, Lenaerts K, Ludolph L, Renman V (2017). Integration of refugees: Lessons from Bosnians in five EU countries. Intereconomics.

[CR3] Battisti M, Giesing Y, Laurentsyeva N (2019). Can job search assistance improve the labour market integration of refugees? Evidence from a field experiment. Labour Economics.

[CR4] Bernhard, I. (2014). *E-government and E-governance: Local implementation of E-government polices in Sweden* [Doctoral dissertation]. KTH Royal Institute of Technology.

[CR5] Bevelander P (2011). The employment integration of resettled refugees, asylum claimants, and family Reunion migrants in Sweden. Refugee Survey Quarterly.

[CR6] Bevelander P (2020). Integrating refugees into labor markets. Iza World of Labor.

[CR7] Bevelander P, Pendakur R (2014). The labour market integration of refugee and family Reunion immigrants: A comparison of outcomes in Canada and Sweden. Journal of Ethnic and Migration Studies.

[CR8] Blix M (2017). Digitalization.

[CR9] Bogic M, Njoku A, Priebe S (2015). Long-term mental health of war-refugees: A systematic literature review. BMC International Health and Human Rights.

[CR10] Böheim, R., & Leoni, T. (2020).* Crisis response monitoring. Austria*. IZA Institute of Labor Economics. Retrieved November 9, 2020, from https://Covid-19.iza.org/crisis-monitor/austria/.

[CR11] Braun V, Clarke V (2006). Using thematic analysis in psychology. Qualitative Research in Psychology.

[CR12] Brell C, Dustmann C, Preston I (2020). The labor market integration of refugee migrants in high-income countries. Journal of Economic Perspectives.

[CR13] Bröckl A, Bliem B (2020). New digital skills. Eine Projektinitiative des AMS.

[CR14] Brorström S, Diedrich A (2020). Boundaries of collaboration—The case of a temporary housing complex for refugees in Sweden. Public Administration Review.

[CR15] Bucken-Knapp G, Fakih Z, Spehar A (2019). Talking about integration: The voices of Syrian refugees taking part in introduction programmes for integration into Swedish society. International Migration.

[CR16] De Vroome T, Van Tubergen F (2010). The employment experience of refugees in the Netherlands. International Migration Review.

[CR17] Delaporte I, Piracha M (2018). Integration of humanitarian migrants into the host country labour market: Evidence from Australia. Journal of Ethnic and Migration Studies.

[CR18] Diedrich A (2017). Validation of immigrants’ prior foreign learning as a framing practice. European Management Journal.

[CR19] Diedrich A, Eriksson-Zetterquist U, Styhre A (2011). Sorting people out: The uses of one-dimensional classificatory schemes in a multi-dimensional world. Culture and Organization.

[CR20] Diedrich A, Hellgren H (2018). Organizing labour market integration of foreign-born persons in the Gothenburg metropolitan area.

[CR21] Emilsson H (2015). A national turn of local integration policy: Multi-level governance dynamics in Denmark and Sweden. Comparative Migration Studies.

[CR22] Esping-Andersen G (1990). The three political economies of the welfare state. International Journal of Sociology.

[CR23] Eurostat. (2020).* Asylum and first time asylum applicants by citizenship, age and sex—Annual aggregated data*. Retrieved November 9, 2020, from https://ec.europa.eu/eurostat/databrowser/view/migr_asyappctza/default/z?lang=en.

[CR24] Falkenhain M, Flick U, Hirseland A, Naji S, Seidelsohn K, Verlage T (2021). Setback in labour market integration due to the Covid-19 crisis? An explorative insight on forced migrants’ vulnerability in Germany. European Societies.

[CR25] Fasani, F., Frattini, T., & Minale, L. (2018). *The struggle for) refugee integration into the labour market: Evidence from Europe* (IZA discussion paper series, 11333). IZA Institute of Labor Economics.

[CR26] Feeney A (2000). Refugee employment. Local Economy.

[CR27] Fossati F, Liechti F (2020). Integrating refugees through active labour market policy: A comparative survey experiment. Journal of European Social Policy.

[CR28] Frödin O, Kjellberg A (2020). Anställningsbidrag: Integration eller etnisk segmentering?. Arbetsmarknad and Arbetsliv.

[CR29] Garcia Alonso, R., Thoene, U. & Dávila Benavides, D. (2020). Social computing applications as a resource for newly arrived refugees in Kronoberg, Sweden.* Digital Policy, Regulation and Governance, 23*(1), 21–44. 10.1108/DPRG-05-2020-0063.

[CR30] Guadagno, L. (2020). *Migrants and the Covid-19 pandemic: An initial analysis* (Migration Research Series, No. 60). International Organisation for Migration.

[CR31] Hagelund A (2020). After the refugee crisis: Public discourse and policy change in Denmark, Norway and Sweden. Comparative Migration Studies.

[CR32] Hunt L (2008). Women asylum seekers and refugees: Opportunities, constraints and the role of agency. Social Policy and Society.

[CR33] Jutvik K, Robinson D (2020). Permanent or temporary settlement? A study on the short-term effects of residence status on refugees’ labour market participation. Comparative Migration Studies.

[CR34] Kavaliunas A, Ocaya P, Mumper J, Lindfeldt I, Kyhlstedt M (2020). Swedish policy analysis for Covid-19. Health Policy and Technology.

[CR35] Knappert L, van Dijk H, Ross V (2020). Refugees' inclusion at work: A qualitative cross-level analysis. Career Development International.

[CR36] Konle-Seidl R (2018). Integration of refugees in Austria, Germany and Sweden: Comparative analysis.

[CR37] Lee ES, Szkudlarek B, Nguyen DC, Nardon L (2020). Unveiling the canvas ceiling: A multidisciplinary literature review of refugee employment and workforce integration. International Journal of Management Reviews.

[CR38] Ortlieb R, Eggenhofer-Rehart P, Leitner S, Hosner R, Landesmann M (2021). Do Austrian Programmes facilitate labour market integration of refugees?. International Migration.

[CR39] Papademetriou DG, Terrazas A (2009). Immigrants and the current economic crisis.

[CR40] Patton M (2015). Qualitative research and evaluation methods: Integrating theory and practice.

[CR41] Phillimore J (2012). Implementing integration in the UK: Lessons for integration theory, policy and practice. Policy & Politics.

[CR42] Ponzoni E, Ghorashi H, van der Raad S (2017). Caught between norm and difference: Narratives on refugees’ inclusion in organisations. Equality, Diversity and Inclusion.

[CR43] Qvist M (2017). Meta-governance and network formation in collaborative spaces of uncertainty: The case of Swedish refugee integration policy. Public Administration.

[CR44] Scholten P (2020). Mainstreaming versus alienation: Conceptualising the role of complexity in migration and diversity policymaking. Journal of Ethnic and Migration Studies.

[CR45] Scholten P, Collett E, Petrovic M (2017). Mainstreaming migrant integration? A critical analysis of a new trend in integration governance. International Review of Administrative Sciences.

[CR46] Scholten P, van Bruegel I (2018). Mainstreaming integration governance. New trends in migrant integration policies in Europe.

[CR47] Swedish Public Employment Service (2020).* Labour market outlook spring 2020 - Summary*. Stockholm: Swedish Public Employment Service. Retrieved July 11, 2021, from https://arbetsformedlingen.se/statistik/analyser-och-prognoser/arbetsmarknadsprognoser/riket/arbetsmarknadsprognos-2020-2021.

[CR48] Swedish Center for Digital Innovation. (2020). *Statusrapport - digital mognad i offentlig förvaltning*. SCDI report.

[CR49] Swedish Agency for Economic and Regional Growth. (2020). *Statistik om korttidsarbete*. Retrieved November 12, 2020, from www.tillvaxtverket.se.

[CR50] Syed J (2008). Employment prospects for skilled migrants: A relational perspective. Human Resource Management Review.

[CR51] Syed J, Özbilgin M (2009). A relational framework for international transfer of diversity management practices. International Journal of Human Resource Management.

[CR52] Takenoshita H (2017). The impact of the recent economic crisis on unemployment among immigrants in Japan. Journal of International Migration and Integration.

[CR53] Verwiebe R, Kittel B, Dellinger F, Liebhart C, Schiestl D, Haindorfer R, Liedl B (2019). Finding your way into employment against all odds? Successful job search of refugees in Austria. Journal of Ethnic and Migration Studies.

[CR54] Vogt IJ (2019). The impact of the financial crisis on European attitudes toward immigration. Computational Management Science.

[CR55] WIFO. (2021). *WIFO-Konjunkturbericht März 2021*. Retrieved March 11, 2021, from https://www.wifo.ac.at/news/news_detail?j-cc-id=1614821557685&j-cc-node=news.

[CR56] Wikström E, Sténs A (2019). Problematising refugee migrants in the Swedish forestry sector. Transfer: European Review of Labour and Research.

[CR57] Wilkinson S, Silverman D (2004). Focus group research. Qualitative research: Theory, method, and practice.

